# Antihypertensive therapy tailored to the circadian blood pressure profile as precision medicine

**DOI:** 10.1038/s41440-026-02789-0

**Published:** 2026-08-28

**Authors:** Francesca Coccina, Chiara Cuccurullo, Rosa Scurti, Sante D. Pierdomenico

**Affiliations:** 1 Department of Innovative Technologies in Medicine & Dentistry, University “Gabriele d’Annunzio” Chieti-Pescara, Chieti, Italy; 2 Geriatrics Unit, Pescara Hospital, Pescara, Italy; 3 Department of Medicine and Ageing Sciences, University “Gabriele d’Annunzio” Chieti-Pescara, Chieti, Italy

**Keywords:** Implemental hypertension, Morning hypertension, Nighttime hypertension, Precision medicine, Chronotherapy

## Abstract

Circadian blood pressure (BP) values may be different from clinic BP values. Thus, antihypertensive therapy guided solely by clinic BP might not prove appropriate. Moreover, ambulatory BP parameters are superior to clinic BP in predicting outcomes, indicating they should receive more attention as therapeutic targets. Among all relevant 24-h BP parameters, in both untreated and treated patients, two clinically unpredictable windows with proven prognostic relevance deserve attention: the period after awakening and the nighttime one. Some patients exhibit higher BP after awakening, which is associated with increased risk. In untreated patients, underdosed therapy could be started. In treated ones, most likely the drug taken the previous morning could not provide adequate duration of action. In this context, a medication with delayed onset of action and peak effect after several hours added in the evening could be of benefit to blunt morning BP rise. Others show high nighttime BP, which is associated with increased risk. In untreated patients, this can lead to prescribe underdosed therapy. In treated patients, most likely antihypertensive therapy in the morning does not cover the 24-h period. In this context, a drug with rapid onset of action and peak effect after few hours added in the evening could reduce high nighttime BP. Globally, evening therapy must be tailored to nighttime BP pattern to achieve the desired effect in the early morning or during the night. Future studies in which antihypertensive therapy is tailored to circadian BP profile, a more physiological definition of chronotherapy, should be scheduled.

**Figure:**
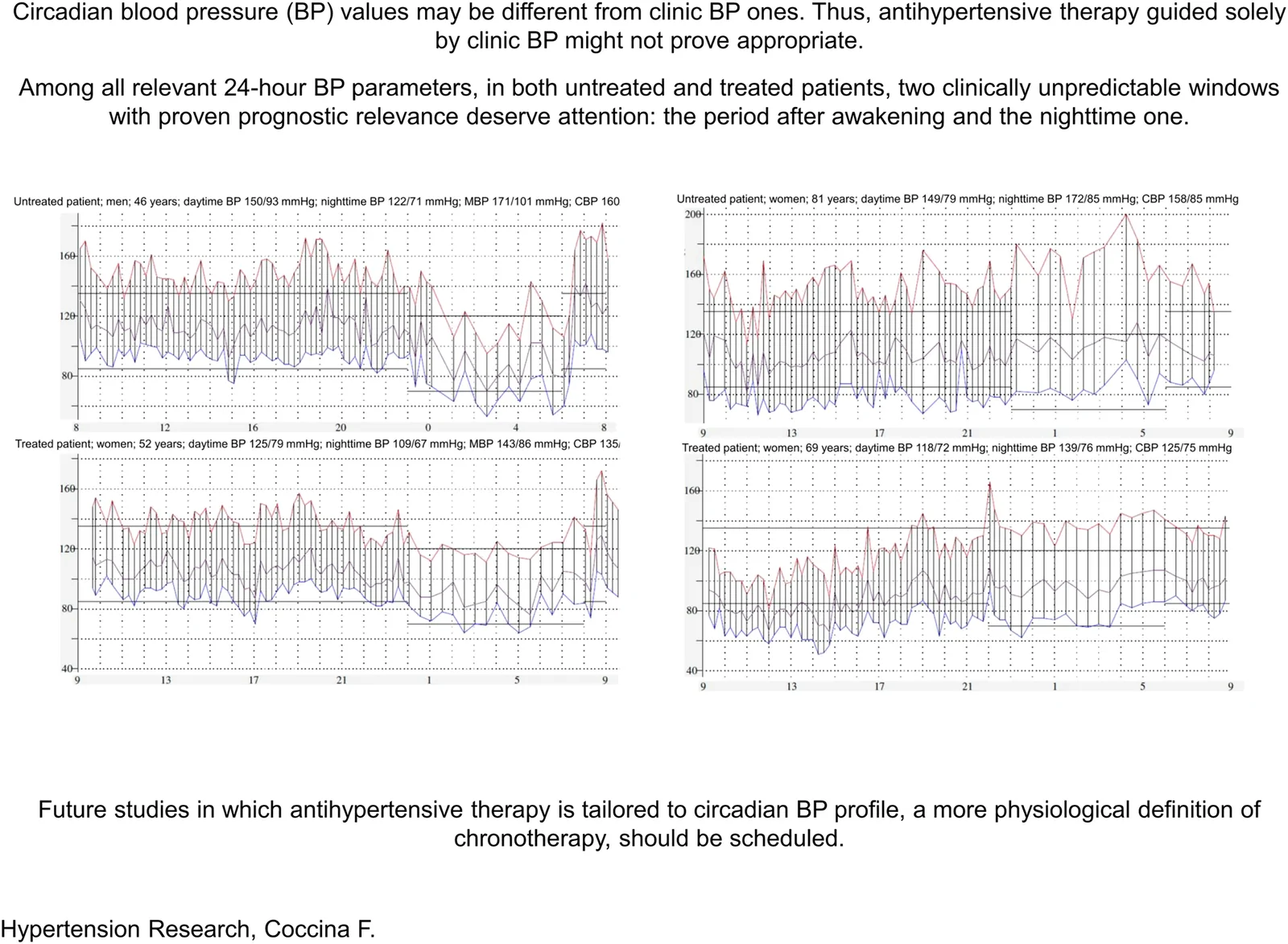

Hypertension is one of the most important modifiable risk factors for stroke, myocardial infarction, heart failure and chronic kidney disease, worldwide [1, 2].

The introduction in the clinical practice of noninvasive 24-h ambulatory blood pressure (BP) monitoring has given us the opportunity to verify that a patient’s BP level during the day and night can differ from that recorded in the clinical setting, and that patients with similar clinic BP readings can exhibit markedly different 24-h BP profiles, including morning BP, morning BP surge, daytime BP, nighttime BP and nighttime BP phenotype, that is, dipping, non-dipping, extreme-dipping and reverse-dipping [1, 2].

In this context, antihypertensive therapy guided solely by clinic BP readings—without knowledge or consideration of the 24-h BP profile—might prove not appropriate and represent a potential cause of suboptimal BP control (hidden or masked uncontrolled hypertension during daytime or nighttime or the 24-h period).

Moreover, it has been widely reported that daytime BP, nighttime BP and circadian BP changes are superior to clinic BP in predicting cardiovascular outcomes in hypertensive patients [3–10]. Concerning daytime and nighttime BP, some studies have described superiority of nighttime BP over daytime BP in the prediction of cardiovascular risk [3, 7, 10]. Globally, this data further reinforces the concept that BP values and its changes during the 24-h period should receive greater attention as therapeutic targets.

For about 20 years, we wondered whether it is better to take antihypertensive medication in the morning or in the evening, or both, to achieve better cardiovascular protection in hypertensive patients. First investigations [11, 12] reported that evening therapy was better than morning one. Recently, two large, randomized trials, that is, the Treatment in Morning versus Evening (TIME) study [13] and the Bedtime versus morning use of antihypertensives for cardiovascular risk reduction (BedMed) study [14] have shown that morning and evening therapy have a similar effect on the occurrence of global cardiovascular events in community resident patients. However, an itemized analysis of the abovementioned studies reveals that morning or evening therapy tended to have a different impact on the various types of outcomes [13, 14]. Particularly, a substantial trend toward reduction in heart failure with evening therapy was observed in both studies [13, 14]. This finding attained statistical significance in a further analysis of the two studies combined [15].

Thus, the best time to take antihypertensive therapy is not yet completely clear. Notably, chronotherapy is an approach that synchronizes pharmacological treatment with the body’s natural biological rhythm (circadian rhythm) to administer therapy when it is most effective and best tolerated. In such a context, given the heterogeneity of the circadian BP profile in hypertensive patients, viewing antihypertensive chronotherapy merely as the administration of treatment exclusively in the morning or exclusively in the evening appears to be an incomplete concept.

Among all relevant BP parameters recorded over the 24-h period—in both untreated and treated patients—there are two clinically unpredictable windows with proven prognostic relevance [3–10]: the period after awakening and the nighttime one.

Some patients, either untreated or treated, exhibit higher BP levels in the hours immediately following awakening (frequently in the context of a dipping or extreme-dipping nighttime BP phenotype), which are associated with higher cardiovascular risk [5, 6, 9]. Thus, in untreated patients an underdosed antihypertensive therapy may be started (Fig. 1, upper panel). In treated patients, most likely the medication taken the previous morning does not actually provide a duration of action of or more than 24 h—or its effect is significantly diminished by that time—while the drug taken on the morning of the same day takes some time to begin working (Fig. 1, lower panel). In this context, one medication with delayed onset of action and peak effect after several hours added in the evening (not multiple medication or those with early peak of action because of a potential detrimental effect in the extreme-dipping phenotype), could be of benefit to blunt the morning rise in BP while waiting for the drug taken in the morning to reach full effect and contribute to control daytime BP.

**Fig. 1:**
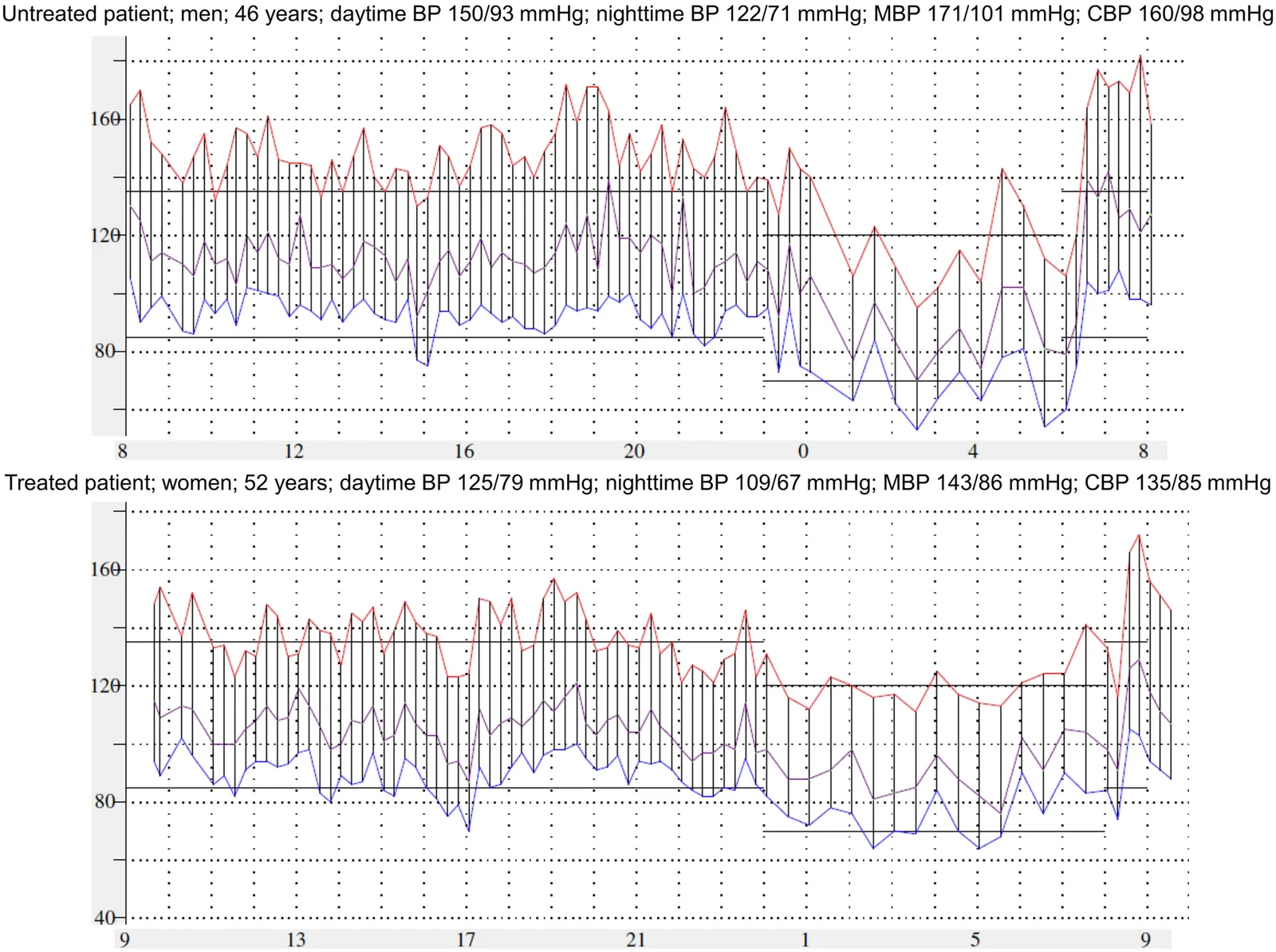
Ambulatory blood pressure (BP) monitoring showing higher morning BP in an untreated (upper panel) and treated (lower panel) patient. CBP clinic blood pressure, MBP morning BP. Values were calculated based on the detailed indications reported in the diary

Some other patients, either untreatd or treated, show high nighttime BP levels (frequently in the context of the non-dipping phenotype) or higher nighttime BP values than daytime ones (reverse-dipping phenotype), which are associated with increased cardiovascular risk [3–8, 10]. In untreated patients, this unpredictable aspect according to clinic BP measurement can lead to the prescription of an underdosed therapy (Fig. 2, upper panel). In treated patients, most likely the antihypertensive therapy taken in the morning may not cover the entire 24-h period because its residual activity may not be sufficient to reduce high nighttime BP values (Fig. 2, lower panel). In such a context, a drug with a quite rapid onset of action and peak effect after few hours added in the evening could be of benefit in patients with high nighttime BP and non-dipping or reverse-dipping phenotypes, in addition to an appropriate morning antihypertensive therapy.

**Fig. 2:**
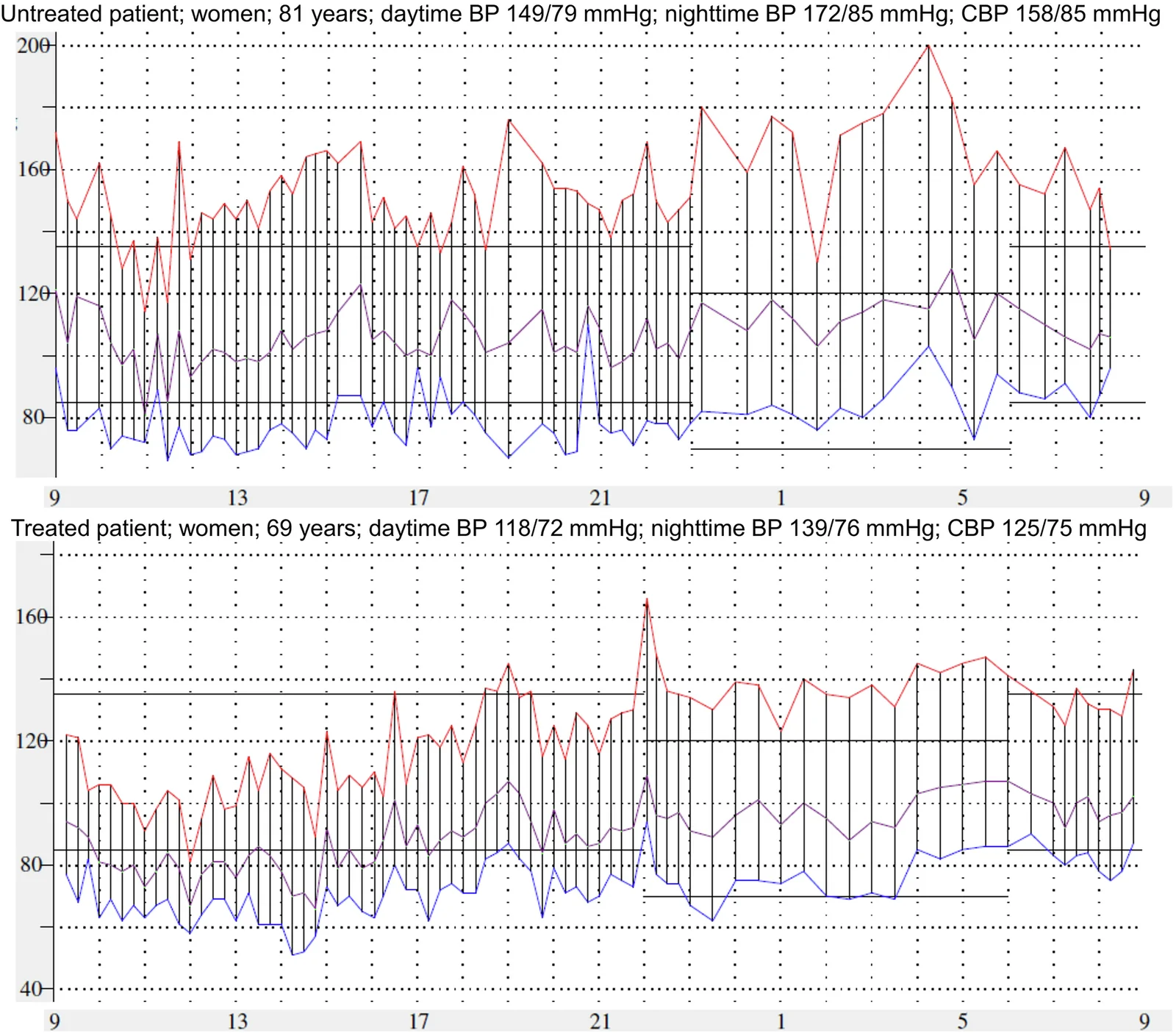
Ambulatory blood pressure (BP) monitoring showing higher nighttime BP in an untreated (upper panel) and treated (lower panel) patient. CBP clinic blood pressure. Values were calculated based on the detailed indications reported in the diary

In a few words, evening antihypertensive therapy must be tailored to nighttime BP pattern using drugs with appropriate pharmacokinetics, so as to achieve the desired effect in the early morning hours or during the night. Although morning antihypertensive therapy might be the starting point, when indicated, we should move away from the paradigm of all antihypertensive therapy in the morning and add a drug in the evening.

It is hoped that future studies will be designed in which antihypertensive therapy is tailored to circadian BP profile by using medications with appropriate pharmacokinetics. This approach would represent a more physiological definition of chronotherapy.
